# Reliability of inertial sensors in the assessment of patients with vestibular disorders: a feasibility study

**DOI:** 10.1186/s12901-017-0034-z

**Published:** 2017-02-02

**Authors:** Sathish K. Sankarpandi, Alice J. Baldwin, Jaydip Ray, Claudia Mazzà

**Affiliations:** 1 INSIGNEO Institute for in silico Medicine, University of Sheffield, Sheffield, UK; 2 School of Health Sciences, University of Salford, Manchester, UK; 3 Ear, Nose and Throat Surgery Department, Sheffield Teaching Hospitals, Sheffield, UK; 4 Department of Mechanical Engineering, University of Sheffield, Sheffield, UK; 5 University of Salford, 111, Crescent House, England M5 4WT UK

**Keywords:** Vestibular disorders, Reliability, OPAL inertial sensors, TUG and sway tests

## Abstract

**Background:**

Vestibular disorders affect an individual’s stability, balance, and gait and predispose them to falls. Traditional laboratory-based semi-objective vestibular assessments are intrusive and cumbersome provide little information about their functional ability. Commercially available wearable inertial sensors allow us to make this real life assessments objective, with a detailed view of their functional abilities. Timed Up and Go (TUG) and Postural Sway tests are commonly used tests for gait and balance assessments. Our aim was to assess the feasibility, test-retest reliability and ability to classify fall status in individuals with vestibular disorders using parameters derived from the commercially available wearable system (inertial sensors and the Mobility Lab Software, APDM, Inc.).

**Methods:**

We recruited 27 individuals diagnosed either with unilateral or bilateral vestibular loss on vestibular function testing. Instrumented Timed Up and Go (iTUG) and Postural Sway (iSway) were administered three times during the first session and then repeated at a similar time the following week. To evaluate within and between sessions reliability of the parameters the Intra-Class Correlation coefficient (ICC) was used. Subsequently, the ability of reliable parameters (ICC ≥ 0.8) to classify fallers from non-fallers was estimated.

**Results:**

The iTUG test parameters showed good within and between sessions’ reliability with mean ICC (between-sessions) values of 0.81 ± 0.17 and 0.69 ± 0.15, respectively. For the iSway test, the relative figures were; 0.76 ± 0.13 and 0.71 ± 0.14, respectively. A retrospective falls classification analysis with past 12 months falls history data yielded an accuracy of 66.70% with an area under the curve of 0.79. Mean Distance from centre of COP (mm) of accelerometer’s trajectory (m/s^2^) from the iSway test was the only significant parameter to classify fallers from non-fallers.

**Conclusions:**

Using a commercially available wearable system a subset of reliable iTUG and iSway parameters were identified and their ability to classify fallers were estimated. These parameters have potential to augment assessments of vestibular patients to enable clinicians and therapists to provide objective, tailored, personalised interventions for their gait and postural control and also to objectively evaluate and monitor the efficiency of their interventions.

## Background

The ability of an individual to maintain posture and balance in both static and dynamic modes is essential for safe, independent living. Vestibular pathology is a common phenomenon frequently seen in the ageing population. Individuals with vestibular pathology commonly present with unsteadiness and imbalance, both of which can lead to falls and fragility fractures [1]. Falls, especially in the elderly, are associated with increased morbidity and can have a negative impact on socialisation resulting in a loss of independence and consequent reduced quality of life [2, 3]. A recent study found that 80% of fallers admitted to Emergency departments in the UK had symptoms of an underlying vestibular impairment [4]. In 2010, the UK spent £203 million treating individuals who had sustained falls highlighting a substantial financial expenditure and significant pressure on the health care services [5]. A prompt, accurate diagnosis and management of the instability of patients with vestibular deficits would reduce the number of fallers and associated morbidity and healthcare expenditure.

Clinical tests of vestibular patients include the Romberg’s test, Tandem Gait, and Fukuda’s stepping tests which tend to be inaccurate and not always reproducible. The Timed up and Go (TUG) test used widely by other specialities is more objective and repeatable and could be used to augment the functional assessment of the vestibular patients.

Vestibular assessments generally available in dedicated balance clinics are laboratory based and include rotation tests, electronystagmography (ENG), videonystagmography (VNG) and occasionally Computerised Dynamic Posturography (CDP) [6]. Most provide an estimate of the function of the vestibular organ whilst only the CDP has the advantage of being able to quantify static balance functions related to posture and stability. However, it is cumbersome and intrusive, as it usually requires the patient to be restrained in a harness whilst the floor is moved and does not reflect the patient’s natural setting [7]. Its cost is not negligible and its availability is limited to only a few tertiary centres. Following these tests most patients will receive vestibular rehabilitation, which may be delayed depending on local availability of resources. Therefore, the need for a simple, portable, quick, objective, reliable and repeatable assessment technique that would allow clinicians to diagnose deficits in real life situations, monitor the rehabilitation progress and predict falls is imperative.

The Timed up and Go (TUG) and postural sway tests used widely by other specialities and could be used to augment the functional assessment of the vestibular patients. Nevertheless, results these tests suffer from subjective assessments. Currently, instrumented version of TUG and Sway tests using inertial sensors show that results of these tests are objective, reproducible and better than traditional assessments [8–12]. Inertial wearable sensors (triaxial gyroscopes and accelerometers) are small, lightweight and inexpensive devices are being increasingly explored to test balance impairments in individuals with neurodegenerative conditions and vestibular impairments [10, 13]. Although research shows that inertial sensors are capable to screen participants with vestibular disorders it is not widely used in the everyday clinical environment. This may be because i) the inertial sensor systems are not yet fully translated for a simple use by a clinician ii) feasibility and reliability of the system in the clinical environment is not well reported.

Recently, commercial exploration of inertial sensors system led to the development of systems that are portable, automated, easy to use, and setup. This preliminary study aims to investigate the feasibility of use and reliability of the one of the commercially available inertial sensor systems known as Mobility Lab™ (APDM, Inc.) [14]. In particular, it aims to assess the test-retest reliability of parameters extracted from instrumented Timed Up and Go (TUG) and Sway tests and also to investigate the ability of reliable parameters to classify fallers in the cohort of participants with vestibular impairments.

## Methods

### Participants

Twenty-seven patients (7 males and 20 females, age range: 40-81 years) were recruited from those that visited the Neurotology Clinics. An informed consent was obtained prior to participation.

Inclusion criteria were:

i) Unilateral labyrinthine weakness and
ii) Bilateral labyrinthine loss, as indicated by their vestibular function tests.

All underwent objective vestibular assessments in the vestibular laboratory using Videonystagmography, Electronystagmography, Caloric testing, and Posturography to measure labyrinthine weakness / failure.

Exclusion criteria were:

i) Active Meniere’s disease and vestibular hydrops,
ii) Benign Paroxysmal Positional Vertigo (BPPV)
iii) History of dizziness related to Transient Ischaemic Attacks (TIAs) due to the fluctuant nature of symptoms.
iv) Individuals with central pathologies including epilepsy, significant visual impairment, excessive alcohol consumption and those on vestibular sedatives were also excluded.

To recruit a homogenous vestibular cohort of participants and exclude every other non-vestibular cause of balance problems, a detailed medical history was undertaken and a full list of concurrent medication was cross-checked for any contribution towards vestibular symptoms and / or falls.

The National Research Ethics committee West Midlands-Edgbaston, reference number 14/WM/0146, approved the study.

### Protocol

The Mobility Lab™ system was used to record, process and store data obtained from previously validated algorithms [10, 12, 14]. The system is composed of six different Magnetic Inertial Measurement Units (MIMUs), each of which contains 3-axis accelerometers (±6 g), 3-axis gyroscopes (±2000°/s for the yaw and roll axes, and ±1500°/s, for the pitch axis) and 3-axis magnetometers (±6 Gauss). MIMU’s were attached around wrists, sternum, chest and shanks of the participants using adjustable Velcro straps. All the sensors were configured for synchronised recording and real-time data acquisition at a sampling rate of 128Hz.

The participants were asked to perform an iTUG and an iSway test following the protocols suggested by the system manufacturer [14]. In order to assess within and between sessions reliability, each patient underwent two sessions, one week apart from each other, and three repetitions of both iTUG and iSway tests were performed in each session.

All patients and operators were asked to fill in a questionnaire on how easy or difficult it was to set up and use the sensors (1: Easy, 2: Slight difficulty, 3: Significant difficulty, 4: Not possible to use). Operators were additionally asked to record the time taken from setup to obtaining the results.

#### iTUG

The TUG is a test widely used the test to assess balance and mobility [15, 16]. In its instrumented version, participants were instructed to rise from a standard armless chair, walk in a straight line for seven metres, turn 180°, return to the chair and sit down. Individuals were requested not to elevate themselves from the chair with their hands when sitting down and standing up and to walk at their normal ambient pace. The tape was used to mark the floor at the seven metres and these marks were clearly shown to the participants. Thirty-two parameters were obtained from the sensor signals. The parameters extracted are grouped based on individual subcomponents of the iTUG task, i.e. the gait, turning, sit-to-stand, turn-to-sit. The details of algorithms used to extract iTUG parameters can be found in [17]. The list of extracted parameters is shown in Table 1 and Appendix.

**Table 1:** Test-retest reliability, mean, Standard Deviation (SD), of parameters with excellent reliability (ICC (2,1) ≥0.8) from iTUG

Parameters	Mean ± SD	Test1	Test2	B/W
		ICC (1,1)	ICC (1,1)	ICC (2,1)
Total Duration (s)	29.67 ± 14.31	0.63	0.98	0.80
Stride Length (m)	1.24 ± 0.15	0.78	0.98	0.81
Stride Velocity (%statures)	62.21 ± 15.10	0.86	0.98	0.82
RoM Shank (deg.)	68.66 ± 10.10	0.93	0.98	0.80
RoM Knee (deg.)	53.78 ± 4.81	0.97	0.93	0.81
Peak Swing Velocity (deg./s)	321.93 ± 59.27	0.94	0.98	0.84
Cadence (steps/min)	99.63 ± 14.23	0.84	0.97	0.83
RoM Trunk horizontal (deg.)	6.29 ± 2.90	0.96	0.92	0.80
Peak Frontal Trunk Velocity (deg./.s)	35.85 ± 13.80	0.92	0.97	0.80

#### iSway

In this instrumented version of a postural sway test, participants were instructed to stand comfortably with their arms folded across their chest. Participants were subsequently instructed to look straight ahead focusing on a fixed target standing still for thirty seconds. Placing a wooden wedge between the patients’ feet ensured consistent foot positioning. Forty-seven parameters were calculated using the signals from the sensor placed on the sternum. The parameters of the iSway are grouped under three categories: jerk, time domain and frequency domain parameters. The details of algorithms used to extract iSway parameters can be found in [10]. The list of extracted parameters is shown in Table 2 and Appendix.

**Table 2:** Test-retest reliability, mean, Standard Deviation (SD), of parameters with excellent reliability (ICC (2,1) ≥0.8) from iSway

Parameters	Mean ± SD	Test1	Test2	B/W
		ICC (1,1)	ICC (1,1)	ICC (2,1)
*Time Domain Parameters*				
RMS sway (ms-2)	0.196 ± 0.17	0.84	0.88	0.83
Mean distance (mm)	0.154 ± 0.13	0.84	0.9	0.83
Mean distance ML (mm)	0.07 ± 0.08	0.85	0.91	0.86
Path length AP (mm)	12.17 ± 12.86	0.89	0.90	0.87
Total sway area (m2/s5)	0.08 ± 0.19	0.78	0.88	0.80
RMS sway ML (ms-2)	0.10 ± 0.13	0.86	0.87	0.86
Path length (mm)	19.86 ± 28.36	0.89	0.93	0.91
Path length ML (mm)	12.85 ± 23.69	0.89	0.94	0.92
Range of acceleration (ms-2)	1.42 ± 1.62	0.84	0.84	0.83
Range of acceleration ML (ms-2)	0.94 ± 1.48	0.84	0.81	0.83
*Jerk Parameters*				
Jerk AP (m^2^/s^5^)	0.16 ± 0.50	0.93	0.84	0.85
Jerk (m^2^/s^5^)	10.08 ± 38.12	0.91	0.86	0.89
Jerk ML (m^2^/s^5^)	0.52 ± 2.07	0.88	0.86	0.88
*Frequency Domain Parameters*				
Mean frequency AP (Hz)	0.6 ± 0.32	0.89	0.81	0.83
Mean frequency (Hz)	0.62 ± 0.30	0.88	0.86	0.84
Total power ((ms-2)2*Hz-1)	13.85 ± 30.11	0.75	0.9	0.80
Total power ML ((ms-2)2*Hz-1)	17.90 ± 51.22	0.86	0.88	0.86
High frequency power AP ((ms-2)2*Hz-1)	18.76 ± 24.10	0.93	0.79	0.80
Low frequency power AP ((ms-2)2*Hz-1)	367.23 ± 78.8	0.93	0.79	0.80

#### Data analysis

To evaluate within and between sessions reliability of the parameters from the iTUG and iSway tests the Intra-Class Correlation coefficient (ICC) was used. In particular, ICC (1,1) was used for within-session reliability, since the inertial unit, the subjects and the assessor remained the same, whereas ICC (2,1) was used for between sessions reliability. The following validated thresholds for ICC values were used: ICC ≥ 0.8 excellent, 0.6 < ICC < 0.8 good, 0.4 ≤ ICC ≤ 0.6 moderate and ICC < 0.4 poor reliability. The ability of parameters to classify falls status based on falls history of a participant was also assessed using self-reported questionnaire of falls in the last 12 months.

Normality of the data was assessed using the Kolmogorov-Smirnov test. A widely used discriminant classifier model with stepwise function was performed to assess the degree to which different parameters were able to identify fall status [16, 18–20] . The discriminant function was based on Mahanolobis distance of each variable and was based on leave-one-out validation method. Statistical analysis was carried out in SPSS v22 and MATLAB (Math works, R2014a), with a level of significance set to *p* = 0.05.

## Results

All patients included in the study were able to complete both the iTUG and the iSway protocols. All experimental sessions lasted less than fifteen minutes and both operator and participant did not encounter any difficulties. Results from both tests, ease of use of the system and falls classification are reported below:

### iTUG

In total, 32 parameters were extracted from the iTUG test. Most parameters demonstrated good reliability (Table 1 and Appendix), with mean ± Standard Deviation (SD) ICC values were :0.81 ± 0.17 for within and 0.69 ± 0.15 for between sessions, respectively. Out of all the parameters, nine showed excellent, sixteen good, five moderate and two poor reliability.

By looking individually at the task components, it was found that the mean ICC (between sessions) values of 0.76 ± 0.05 for the gait component, 0.62 ± 0.10 for the turning component 0.31 ± 0.08 for the sit-to-stand component, and 0.67 ± 0.07 for the turn-to-sit component, respectively.

### iSway

Forty-seven parameters were extracted from the iSway test (Table 2 and Appendix). The mean ICC values of iSway parameters for within and between sessions were 0.76 ± 0.13 and 0.71 ± 0.14, respectively. More specifically, twenty-seven showed excellent, fifteen good, three moderate and two poor reliability values. Mean ICC (between sessions) values of the individual category of parameters of iSway were: 0.76 ± 0.14 for the time domain parameters, 0.68 ± 0.13 for the frequency domain parameters and 0.76 ± 0.12 for the jerk component.

### Association with falls history

We explored the added value of parameters from both the tests to classify retrospective falls status of participants in the past 12 months.

In the selection process, we only included parameters which had excellent reliability. Nineteen parameters from iTUG and eighteen parameters from iSway had excellent reliability (shown in Tables 1 and 2). All 37 parameters with excellent reliability were added as an input to the classifier. Interestingly, none of the parameters from iTUG was significant and therefore excluded, only ‘Mean distance of the COP (mm)’ from iSway (*p* = 0.036) was selected by the classifier.

**Fig. 1:**
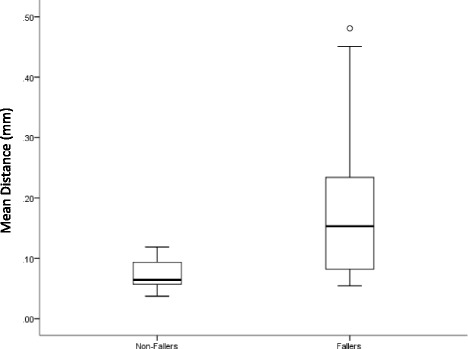
Boxplot of Mean distance from the COP of fallers and non-fallers

Table 3 shows the summary of classification performance of the classifier. The sensitivity of the classifier was 55%, specificity was 100%, overall accuracy was 66.67% and with an area under the curve of 0.79. Boxplot of Mean distance from COP is shown in Fig. 1.

**Table 3:** Confusion matrix of the classifier

	Fallers	Non-fallers
Fallers	11	9
Non-fallers	0	7

### Sensors’ ease of use and timing

The questionnaires on ease of use were completed by all twenty–seven participants and five operators (operators included were an engineer, physiotherapists, a consultant and a junior doctor) on a scale from to 1 to 4 (1 being easy and 4 not possible to use), they all gave a score of 1.

The average time recorded to set up equipment was five minutes with tests taking on average ten minutes followed by a two-minute download period at the end of the each assessment.

## Discussion

The aim of this small study was to investigate the feasibility of using a commercially available wearable inertial sensor system in a busy clinical environment and to assess the reliability of the gait and balance parameters from the system in order to set up a larger study. A commercial wearable system known as OPAL APDM Inc. with the associated Mobility Lab™ software was used to acquire and process the data.

An instrumented version of two widely used tests to assess gait and balance function, the TUG and Sway tests, was used to test the patients’ walking and balance ability. The ability of the patients to perform these tests is usually evaluated using score associated with ordinal scales, but the addition of the sensors provides more objective and reliable results [8, 12, 21, 22].

The main contribution of the study was to assess the reliability of extracted parameters robustly and investigate the potential for fall classification. Most of the previous studies have only investigated the within-day reliability of iTUG and iSway parameters [10, 12]. This study besides the within-day reliability, between-day reliability was also assessed by looking at test-retest data collected one week apart from each other at similar times during the day to ensure the consistency of results of the commercial system.

Several reliable parameters from both the tests and its subcomponents were identified. In particular, the parameters from gait sub-component of the iTUG test were most reliable. ‘Peak swing velocity (deg. /s)’ of the lower limb and ‘Cadence (steps/min)’ of gait showed highest between-sessions ICC values of 0.84 and 0.83 respectively. These results are consistent with other studies [12, 17]. The only parameters with ICC(2,1) greater than 0.80 were: Mean distance mediolateral (ML), Path length anterior-posterior (AP), Root mean square sway ML, Path length and Path length ML whereas the frequency domain parameters were: Total power ML were most reliable, with ICC values greater than 0.85.

The least reliable part of the iTUG test was the sit-to-stand component, which is also been reported in the different populations [23]. A potential cause for poor reliability is the minimum number of constraints imposed on the participants (i.e. use vs. no use of their arms) allowing them to perform the test in a number of different ways. Another reason could be associated with the positioning of the sensors, since even a slight change in their orientation might affect the amplitude of the recorded acceleration and angular velocity signals, and hence the values of the extracted parameters [11].

The parameters ‘Path length of sway’ the total length of COP trajectory [mm] ([m/s2]) in the mediolateral direction (ML) and ‘Jerk’ (m^2^/s^5^) the sway jerkiness showed highest reliability values. These results are similar to findings from Mancini et al. [7] on patients with untreated Parkinson's disease. On comparison with iTUG, The parameters calculated for the iSway demonstrated an overall lower reliability, that may be due to the short period taken to complete the test (30 s) [24]. This study was conducted in the view for potential study with large population size and we propose to extend the iSway duration to 60 s.

It is unsurprising to see that overall within-session reliability ICC (1,1) of Test 2 is higher than Test 1. This is due to the participants becoming familiar with the test' administered and were able to perform it with more consistency.

In addition to reliability, the study assessed the ability of reliable parameters to classify fallers. The parameter ‘Mean distance from the centre of COP trajectory (mm)’ from iSway had a significant ability to classify falls status of the participant. Sensitivity and accuracy of the classifier is moderate and this may be due to insufficient information to robustly classify fallers. Although many of the iTUG parameters had excellent reliability they did not have statistical significance to be included in the classification model. Some of the iTUG parameters had a *p*-value slightly over 0.05 set for the study and were excluded by the classifier, this may be due to a lower number of participants in the study. With the larger sample size, it is highly likely that a number of iTUG parameters will be included by the classifier which in turn will increase accuracy and sensitivity of the classification. This is only an initial feasibility study. So only generic falls episodes have been recorded. Falls episodes have other contributors such as environmental, medication use etc. in addition to the vestibular cause. Future studies will accurately record comprehensive falls episodes prospectively.

Despite the limitations, this study showed that it is feasible to use the inertial sensors for the assessment of patients with vestibular disorders in a busy clinical environment. The results of the study show that the system is easy, quick to setup and use in the busy clinical environment. Participants and operators did not find any issues using the system. Most importantly, several parameters from the system have good to excellent reliability. In addition, this study showed that parameters from Mobility lab™ system can retrospectively classify falls in these patients in almost 70% of the case. This commercially available system has the potential for a use in a clinical environment and beyond with its capabilities of portability.

## Conclusions

Use of inertial sensors allows for an objective assessment of gait, stability, balance and functional abilities of an individual both in their natural and the clinical environment. The wearable sensors are non-intrusive, reliable, user-friendly and provide valuable information on the functional ability of vestibular patients. This study used one of the commercially available sensor system known as Mobility Lab™ and investigated its feasibility, test-retest reliability in a neurotology clinics. The results show that it is easy to setup and feasible to use in clinical settings. The parameters of the system showed good to excellent reliability and a potential role in falls prediction. These preliminary results show an excellent potential towards more personalised and targeted interventions with an objective evaluation of their efficiency. Future longitudinal, prospective studies will focus on testing the validity and sensitivity of the chosen parameters to actually discriminate amongst individuals from within the same patient group and their correlation to the risk of falls.

## Appendix

**Table 4:** Test-retest reliability, mean, Standard Deviation (SD), of all other parameters from iTUG

Parameters	Control group				Patients group			
	Mean ± SD	Test1	Test2	B/W	Mean ± SD	Test1	Test2	B/W
		ICC	ICC	ICC		ICC (1,1)	ICC	ICC
		(1,1)	(1,1)	(2,1)			(1,1)	(2,1)
Stride velocity (m/s)	1.35 ± 0.13	0.96	0.95	0.88	1.04 ± 0.19	0.77	0.98	0.77
Double support (%)	20.66 ± 3.16	0.89	0.87	0.68	27.09 ± 7.70	0.71	0.96	0.70
Swing (%)	39.67 ± 1.58	0.89	0.87	0.68	36.45 ± 3.85	0.71	0.96	0.70
Stance (%)	60.33 ± 1.58	0.89	0.87	0.68	63.55 ± 3.85	0.71	0.96	0.70
RoM trunk frontal (deg.)	17.11 ± 8.34	0.99	0.99	0.62	9.58 ± 3.40	0.92	0.87	0.71
Gait cycle time(s)	1.12 ± 0.10	0.98	0.96	0.90	1.24 ± 0.23	0.66	0.96	0.78
Peak arm swing velocity (deg./s)	167.91 ± 42.7	0.89	0.84	0.79	148.79 ± 55.55	0.8	0.92	0.71
RoM arm (deg.)	21.23 ± 8.84	0.89	0.92	0.68	18.85 ± 9.06	0.9	0.92	0.78
RoM trunk sagittal (deg.)	6.27 ± 2.24	0.92	0.97	0.40	5.00 ± 1.64	0.89	0.92	0.75
Peak horizontal trunk velocity (deg./s)	20.34 ± 7.20	0.96	0.93	0.83	25.89 ± 11.34	0.98	0.9	0.71
Peak sagittal trunk velocity (deg./s)	30.47 ± 5.94	0.89	0.90	0.67	26.95 ± 12.21	0.91	0.97	0.67
Peak frontal trunk velocity (deg./.s)	45.02 ± 14.15	0.98	0.97	0.75	35.85 ± 13.80	0.92	0.97	0.80
*Turning*								
Turn duration (s)	1.93 ± 0.36	0.60	0.71	0.41	3.48 ± 1.85	0.58	0.92	0.70
Turn number of steps	3.78 ± 0.80	0.38	0.24	0.21	5.36 ± 1.91	0.48	0.75	0.58
Turn peak velocity(deg./s)	171.69 ± 26.3	0.45	0.70	0.37	114.12 ± 36.46	0.81	0.91	0.75
Turn step time (s)	0.63 ± 0.09	0.61	0.36	0.53	0.79 ± 0.28	0.59	0.72	0.55
Turn step time before turn (s)	0.57 ± 0.05	0.88	0.82	0.81	0.66 ± 0.18	0.46	0.84	0.52
*Sit to stand*								
Duration (s)	2.30 ± 0.72	−0.03	0.26	0.04	2.72 ± 1.62	0.17	0.27	0.26
Peak velocity (deg./s)	97.45 ± 24.13	0.52	0.72	0.54	91.29 ± 38.21	0.44	0.41	0.26
RoM trunk (deg.)	38.01 ± 7.81	0.51	0.72	0.61	32.61 ± 9.52	0.55	0.81	0.40
*Turn to sit*								
Turn to sit Duration (s)	3.89 ± 0.72	0.59	0.63	0.48	5.21 ± 2.13	0.6	0.88	0.72
Turn to sit RoM trunk (deg.)	24.97 ± 8.55	0.90	0.82	0.58	24.60 ± 7.86	0.71	0.77	0.59

**Table 5:** Test-retest reliability, mean, Standard Deviation (SD), of all other parameters from iSway

Parameters	Control group				Patients group			
	Mean ± SD	Test1	Test2	B/W	Mean ± SD	Test1	Test2	B/W
		ICC	ICC	ICC		ICC (1,1)	ICC	ICC (2,1)
		(1,1)	(1,1)	(2,1)			(1,1)	
*Time domain parameters*								
RMS sway AP (ms-2)	0.066 ± 0.032	0.63	0.64	0.55	0.157 ± 0.13	0.82	0.87	0.77
Mean distance AP (mm)	0.053 ± 0.026	0.64	0.67	0.56	0.121 ± 0.09	0.84	0.86	0.79
@95circle sway area (m2/s4)	0.060 ± 0.071	0.56	0.25	0.27	0.70 ± 1.36	0.70	0.86	0.73
@95ellipse sway area (m2/s4)	0.033 ± 0.039	0.53	0.24	0.28	0.48 ± 0.91	0.65	0.80	0.70
Mean velocity AP (ms-1)	0.131 ± 0.091	0.35	0.29	0.30	0.24 ± 0.23	0.46	0.67	0.41
Mean velocity (ms-1)	0.141 ± 0.092	0.42	0.31	0.34	0.28 ± 0.25	0.50	0.79	0.48
Mean velocity ML(ms-1)	0.035 ± 0.023	0.20	0.39	0.26	0.10 ± 0.10	0.63	0.66	0.55
Range of acceleration AP (ms-2)	0.348 ± 0.218	0.43	0.07	0.28	0.95 ± 0.81	0.78	0.82	0.73
*Jerk Parameters*								
Normalized jerk APAD	1.143 ± 0.293	0.38	0.43	0.42	1.34 ± 0.54	0.80	0.71	0.73
Normalized jerk AD	4.89 ± 1.096	0.43	0.45	0.45	5.34 ± 1.84	0.78	0.78	0.74
Normalized jerk MLAD	2.15 ± 0.663	0.54	0.63	0.49	1.82 ± 0.53	0.62	0.61	0.56
*Frequency Domain Parameters*								
Mean frequency ML (Hz)	1.142 ± 0.416	0.70	0.76	0.57	1.03 ± 0.39	0.82	0.84	0.78
Centroidal frequency AP (Hz)	0.595 ± 0.242	0.37	0.59	0.49	0.7 ± 0.39	0.90	0.81	0.77
Centroidal frequency ML(Hz)	1.163 ± 0.363	0.72	0.8	0.51	1.08 ± 0.35	0.71	0.70	0.67
Frequency dispersion AP AD	0.801 ± 0.054	0.17	0.07	0.19	0.78 ± 0.07	0.61	0.61	0.55
Frequency dispersion AD	0.762 ± 0.048	0.35	0.13	0.07	0.74 ± 0.06	0.37	0.30	0.26
Frequency dispersion ML AD	0.705 ± 0.095	0.50	0.57	0.39	0.68 ± 0.10	0.65	0.75	0.65
@95frequencyAP (Hz)	1.444 ± 0.612	0.39	0.76	0.54	1.60 ± 0.76	0.88	0.8	0.68
@95frequency (Hz)	1.856 ± .557	0.36	0.72	0.52	1.99 ± 0.62	0.73	0.67	0.60
@95frequencyML (Hz)	2.601 ± 0.523	0.70	0.80	0.52	2.39 ± 0.56	0.64	0.60	0.55
Total power AP ((ms-2)2*Hz-1)	1.792 ± 1.950	0.55	0.61	0.38	16.03 ± 26.80	0.73	0.55	0.62
Median frequency AP (Hz)	0.348 ± 0.162	0.16	0.01	0.22	0.44 ± 0.34	0.6	0.73	0.68
Median frequency (Hz)	0.425 ± 0.105	0.32	0.12	0.25	0.49 ± 0.16	0.63	0.45	0.51
Median frequency ML (Hz)	0.719 ± 0.419	0.54	0.59	0.39	0.74 ± 0.43	0.63	0.75	0.66
High frequency power ML((ms-2)2*Hz-1)	54.059 ± 21.90	0.68	0.78	0.60	38.39 ± 26.50	0.93	0.85	0.74
Low frequency power ML ((ms-2)2*Hz-1)	251.92 ± 71.5	0.67	0.78	0.59	303.49 ± 86.9	0.93	0.85	0.74

## Abbreviations

BPPV: Benign paroxysmal positional vertigo
CDP: Computerised dynamic posturography
COP: Centre of pressure
ENG: Electronystagmography
ICC: Intraclass correlation coefficient
Isway: Instrumented sway
iTUG: Instrumented Timed Up and Go
MIMUs: Magnetic inertial measurement units
ML: Mediolateral
SD: Standard deviation
VNG: Videonystagmography.
